# Effects of Introvision, a self-regulation method with a mindfulness-based perception technique in migraine prevention: a monocentric randomized waiting-list controlled study (IntroMig Study)

**DOI:** 10.1186/s10194-023-01684-0

**Published:** 2023-11-03

**Authors:** Monika Empl, Sonja Löser, Petra Spille, Agnieszka Rozwadowska, Ruth Ruscheweyh, Andreas Straube

**Affiliations:** 1 Department of Neurology, Ludwig-Maximilians-University Munich, Munich, Germany; 2 Practice Munich, Munich, Germany; 3 Introvision E.V, Hamburg, Germany; 4 Department of Neurology, Kbo Hospital, Haar, Germany

**Keywords:** Migraine prevention, Introvision, Mindfulness, Self-efficacy

## Abstract

**Background:**

Migraine is a brain disorder with recurrent headache attacks and altered sensory processing. Introvision is a self-regulation method based on mindfulness-like perception techniques, developed at the University of Hamburg. Here, we examined the effect of Introvision in migraine prevention.

**Methods:**

Migraineurs with at least five headache days per month were block-randomized to the experimental group (EG) or waiting list group (WL), the latter starting Introvision training six weeks after the EG. Participants learned Introvision in six weekly on-site group sessions with video-conference support followed by three individual video-conference sessions. Headache diaries and questionnaires were obtained before Introvision training and three months after the last individual Introvision session.

**Results:**

Fifty-one patients completed the study. The primary outcome, headache days of the EG after Introvision training compared to those of the WL before the training, showed no significant effect (10.6 ± 7.7, *n* = 22; vs. 10.9 ± 6.3, *n* = 29, *p* = 0.63; Mann–Whitney-U-Test). The secondary outcome, comparing pooled EG and WL data before and after Introvision training, revealed a significant reduction of headache days (from 11.7 ± 6.5 to 9.8 ± 7.0; *p* = 0.003; Wilcoxon-paired-Test) as well as of acute medication intake and Headache-Impact-Test 6 (HIT-6) scores and increased self-efficacy as quantified by increased FKMS-scores (FKMS: german short form of the Headache Management Self-Efficacy Scale (HMSE)).

**Conclusion:**

Although the study did not reach its primary endpoint, several secondary outcome parameters in the pooled (non-controlled) pre-post analysis showed an improvement with a decrease in monthly headache days by 1.9 days/ month. A larger randomized controlled trial has to corroborate these preliminary findings.

**Trial registration:**

NCT03507400, Registration date 09.03.2018.

**Supplementary Information:**

The online version contains supplementary material available at 10.1186/s10194-023-01684-0.

## Background

Migraine is a brain network disorder with recurrent debilitating headache attacks and altered sensory processing [1]. Sensitivity to light and sound during attacks are part of the diagnostic criteria of migraine. But even outside attacks, altered sensory processing is shown by experimental proof of decreased habituation to repetitive stimuli [2] or altered connectivity between primary and secondary sensory cortices [3]. Moreover, sensory stimuli such as bright light, noise or odors can trigger attacks [4], and olfactory training can reduce attack frequency in children and adolescents and normalize reduced pain thresholds [5].

As stress is a trigger for attacks in about 70–80% of migraineurs [4], stress reduction is an acknowledged method of migraine prevention [6]. Introvision, developed by Angelika C. Wagner, University of Hamburg, is a self-regulation method for stress reduction with a unique mindfulness-based perception technique. As migraineurs have an altered sensory processing, a stress reduction method based on a perception technique might prove effective in migraine prevention. The casual observation of positive effects of Introvision by several migraineurs prompted the conception of this single center randomized waiting-list controlled study.

### Introvision

Introvision is a mental and emotional self-regulation technique aiming to reduce stress and in-duce calm by resolving inner conflicts. It has been validated in over 40 years in a wide range of fields such as tinnitus [7], chronic muscle tension [8], mental blockages in female management trainees [9], sleep quality, competitive sports [10], and many more. According to the theory of Introvision, inner conflicts arise when individual core beliefs or inner demands, collide with the perceived reality. In this case, stress, anxiety or agitation occurs. According to Aaron Beck, negative core beliefs often circle around the three main conditions helplessness, lovelessness, worthlessness [11]. Introvision first identifies core beliefs underlying inner conflicts using a perception technique called "stating attentive perception" (SAP) which aims to reduce or eliminate perceptual filters. Applying SAP has a calming effect, even in the presence of internal conflicts. Then, similar to trauma therapy, Introvision gradually enables to look calmly at the individual’s unpleasant feelings or anxieties. The primary aim of Introvision is to detach the automatic link of negative emotion from the cognition. An elaborated explanation of Introvision can be found in the Supplementary material, see also [12].

Previous studies have shown calming effects of mindfulness-based relaxation techniques and their effectiveness in migraine prevention [6, 13]. Neurophysiologically it has been shown that perception techniques similar to Introvision quickly reduce the activity of the left amygdala, which fits well with the calming effect [14].

## Methods

Adult migraineurs with at least five headache days per months were recruited from the outpatient headache clinic of the Department of Neurology, Ludwigs-Maximilians-University Munich, from the headache praxis of ME, and by Google advertisements from September 2017 to September 2019. The diagnosis of migraine according to ICHD-3 criteria [15] was made or confirmed by ME after history taking and clinical examination. The study was approved by the local ethics committee (N° 632–15) and registered with clinical trials (N° NCT03507400).

Concomitant medication overuse headache and/or episodic tension type headache was allowed, but patients with other primary or secondary headache or facial pain disorders were excluded. Further exclusion criteria were: clinically significant depression (according to 13 or more points in Beck Depression Inventory-Fast-Screen (BDI-FS)) [16], active psychosis, drug addiction (benzodiazepines, opiods), change in headache preventative medication or non-medication preventative measures such as physical activities or acupuncture during the study period. After giving informed consent, the participants were block-randomized (in blocks of 10 each via random-number function of Excel) to the experimental group (EG) or waiting list group (WL), the latter starting Introvision training six weeks after the EG. The waiting-list design was chosen because Introvision training cannot be performed in a blinded manner. A total of 79 participants were included in the study. 7 dropped out before starting Introvision training, 20 discontinued later during the study. The analysis is based on 51 subjects of which 49 provided complete data before and after the introvision training.

Participants learned Introvision in six weekly on-site group sessions with video-conference support by two experienced Introvision supervisors (SL and PS) followed by three individual video-conference sessions by SL or PS. Headache parameters (captured by headache diaries and questionnaires) were compared between the 30 days before the start of Introvision training and days 90–120 after the last individual Introvision session. Headache parameters comprised headache days per month, days with acute attack medication per months, headache intensity (1 mild, 2 moderate, 3 strong), all documented in the standardized headache diaries of the outpatient headache clinic of the Neurology department, as well as questionnaires (HIT-6 [17] and FKMS: “Fragebogen zum Kopfschmerzmanagement und zur Selbstwirksamkeit”, a German translation of the Headache Management Self-Efficacy Scale (HMSE), short form [18]).

At the end of the study, patients were asked for side effects and if they would recommend Introvision for other migraine patients (yes/no).

The mean time between study inclusion to start of group sessions was 61 ± 68 days (median 42 days, range 0- 231 days) for the EG, for the WL (minus the 42 days delay for the waiting list to be comparable) 68 ± 53 days (median 63 days, range 0- 189 days). The average time between start of the group sessions and the evaluated month was 181 ± 27 days (median 178, range 176 to 236 days) for the EG, for the WL 176 ± 23 days (median 167, range 141 to 240 days), as some participants had a delay between the end of group sessions and the last individual sessions for various reasons.

Statistical analysis was performed using SPSS, version 21. As the data were not normally distributed (Kolmogorov–Smirnov-Test), non-parametric statistical tests were used, for unpaired data sets the Mann–Whitney-U Test, for paired data sets the Wilcoxon-paired Test, each two-sided and with the level of significance set at 0.05. The analysis was based on 51 patients for the primary outcome, the waiting list comparison, and on 49 patients (or less) for the pooled analysis of paired secondary outcomes, see also Table 1 and the Consort Flow Diagram. Reasons for dropouts were various, drop out due to start of a CGRP antibody was indicated by 4 participants, but might not have been disclosed in every case.

**Table 1:** Characteristics of the study population

	n	Female / male	Age (years)	Duration of migraine (years)	On preventative medication
EG	22	22/0	44.5 ± 13.6	26.6 ± 13	6^a^
WL	29	25/4	40.7 ± 10.9	22.4 ± 13	4^b^

Values given as mean ± standard deviation

^a^botulinumtoxin: *n* = 2; magnesium *n* = 1, betablocker: *n* = 1; betablocker and magnesium *n* = 1; betablocker and amitriptylin and topiramat: *n* = 1

^b^botulinumtoxin and venlafaxin: *n* = 1, magnesium *n* = 1, betablocker: *n* = 1; CGRP-Antibody: *n* = 1

## Results

Data from 51 participants were analysed, 49 of which provided data before and after Introvision. The primary outcome of the waiting-list controlled part of the study, headache days per month of the EG after Introvision training (10.6 ± 7.7; *n* = 22) compared to those of the WL before Introvision training (10.9 ± 6.3, *n* = 29), showed no significant effect (*p* = 0.63, Mann–Whitney-U Test, see also Table 2). The secondary outcome, stemming from analysis of the non-controlled pre-post part of the study, comparing pooled EG and WL data before and after Introvision training, showed a significant reduction of headache days (by 1.9 days per month), as well as of medication intake and HIT-6 scores, and an increase in self-efficacy. Pain intensity did not change (Table 3). 21.5% (11 of 51) of the participants had an at least 50% reduction of headache days (i.e. were 50% responders) as possible comparative parameter for other headache studies. 27% (13 out of 49) patients had a meaningful reduction of HIT-6 score of ≥ 5 points.

**Table 2:** Primary outcome parameters, WL-controlled part of the study

**Primary outcome**	**Before Introvision training**	**After Introvision training**	
Headache days/month (m) EG	12.7 ± 6.6 *n* = 22	**10.6 ± 7.7** *n* = 22	***p* = 0.63** **Mann–Whitney-U Test**
Headache days/month (m) WL	**10.9 ± 6.3** (*n* = 29)	9.0 ± 6.4 (*n* = 27)

**Table 3:** Secondary outcome parameters, non-controlled pre-post part of the study

**Secondary outcome:** **Pooled EG/WG data**	**Before Introvision training**	**After Introvision training**	
**Headache days/m**	11.7 ± 6.5	9.8 ± 7.0	*n* = 49, *p* = 0.003 Wilcoxon-paired Test (WPT)
**Acute medication days/m**	6.3 ± 3.6	5.0 ± 3.7	*n* = 47, *p* = 0,004, (WPT)
**HIT-6 score (36–78)**	64.3 ± 4.2	61.4 ± 5.9	*n* = 47, *p* < 0.001 (WPT)
**Self-efficacy** **(FKMS-SF) score (6–42)**	21.7 ± 7.7	26.2 ± 6.0	*n* = 46, *p* < 0.001, (WPT)
**Headache intensity** **(1–3)**	2.0 ± 0.5	2.0 ± 0.5	*n* = 49, *p* = 0.376 (WPT)

Values given as mean ± standard deviation

28% (13 of 46) participants stated that they were capable to avert beginning attacks using Introvision. 97% of the participants stated they would recommend Introvision to other migraine patients (45 of 46 answers).

One side effect was documented (a case of self-limiting tachycardia during a SAP (Stating attentive perception) exercise).

### Exploratory analysis

When analysing individual Introvision sessions, it was remarkable that negative core beliefs of many of the migraine patients were centered around helplessness.

## Discussion

Main result of our study is the reduction of headache days per months in the pooled analysis of all participants after Introvision in the open, non-controlled study part. As there were only very limited side effects, our data suggest that Introvison might be a well tolerated non-drug preventative for migraine patients with additional benefits with regard to self-efficacy, an important feature for headache patients, and for stress reduction in general. However, a randomized controlled trial has to corroborate these preliminary findings.

Unfortunately, our study did not reach its primary endpoint, as the number of headache days in the EG after Introvision training was not significantly less than in the WL before training. We believe that this is at least partially due to an imbalance between groups, as the EG had more headache days already before Introvision than the WL before Introvision. In contrast, in the open, non-controlled part of the study nearly all parameters in the secondary, pooled group analysis—apart of headache intensity – were improved after Introvision training. The effect on headache days per month was of moderate size with an average reduction by 1.9 days per month after training, as expected for a non-medication intervention, although we had hoped for a stronger effect based on our previous experience with single migraine patients. A recent study found a similar reduction of 1.6 days per month for MBSR training, with no significant difference from the control group with headache education (-2.0 days) [19]. Similarly, another recent study on MBSR in migraine showed a significant reduction of headache days per month at week 10 (-1.0 days) and at week 20 (-1.4 days) compared with stress management of headaches [13], but no significant reduction at week 52. In patients with chronic migraine and medication overuse headache, addition of a mindfulness training to standard care was clearly superior compared to standard care alone in a randomized controlled trial [20]. Nevertheless, as our patients were strongly affected by their migraine with a median HIT-6 score of over 60, and a long migraine history (mean duration over 20 years), a reduction of 1.9 headache days per month together with the significant reduction of medication days and HIT-6 sores seems meaningful. The 50% responder rate of 21.5% is lower than in a CGRP-antibody trial as expected (for example 29.9% in the LIBERTY study with Erenumab [21]), but underscores the possibly meaningful relief for a considerable number of the patients. Compared to other common prevention methods such as progressive muscle relaxation (PMR) the supposed effect of Introvision seems comparable as PMR reduced migraine days per month by 2.4 days after three months (3.1 days per months compared to 5.5 days before PMR [22]), whereas in general relaxation methods are ascribed an effect of about 35–45% reduction of migraine days [23].

The observed improvement of self-efficacy in headache management was to be expected in an effective self-regulation technique. It ought to be considered that this study is the first in which SAP was taught over videoconference. Although the participants may have learned the method differently from those learning in conventional courses, there is no evidence that this affects the quality of the subsequent implementation in their everyday life.

The dropout rate / discontinuation rate (35%; 28 of 79 randomized participants; 29%; 21 of 72 who started the group sessions) was higher compared to other non-pharmaceutical trials and contributed to a rather low number of participants available for analysis: a meta-analysis showed that 40% of non-pharmacological interventions have a drop out rate of less than 5%, 10% have a drop out rate of over 20% rate, whereas internet-based cognitive behavioral settings show an even higher drop out rate (29–56%) [6]. The need to wait for the start of the group sessions during the sometimes slow recruitment may have increased early drop out rates, as patients may have sought earlier treatment. Additionally, our trial was carried out during the commercial launch of the highly effective and also in the public press discussed CGRP-antibody substances as migraine preventative, so our highly affected patients may have preferred this new option of migraine prevention.

Our choice of primary endpoint might be a matter of criticism. As our intervention could not be applied in a blinded fashion, we decided for a waiting list design. Usually, the primary outcome variable of the two groups, EG after intervention and WL before intervention, are measured at the same time. However, effects of Introvision take approximately three months to become noticeable, as we know from our experience and other studies [8]. As we anticipated that a waiting period of more than three months (in addition to the gap to the course start due to consecutive recruiting) would lead to a very high drop out rate, we settled on a waiting list period of 6 weeks after the start of the corresponding EG as a compromise between feasibility and having a control group. The same applies for the observation period of three months rather than a more desirable six months after introvision, which we deemed not feasible for the same reasons. Unfortunately, as discussed above, the waiting list design was challenged by the baseline group difference in headache days per month before Introvision. This could have been improved by stratifying randomization according to headache frequency.

In future, a “virtual” control group could solve this dilemma, by recruiting age-, sex-, and headache-impact-matched controls without a change of treatment from an online-headache-registry, such as the “DMKG Headache Registry” [24], the headache registry of the German migraine and headache society. Thus, a future, larger, randomized controlled study of effects of Introvision should use headache-frequency stratified control groups, and a prolonged observation period of six months, as the effects of Introvision might become fully manifest only at that time.

It was remarkable, that individual negative core beliefs of many of the migraine patients were centered around helplessness. Helplessness can easily be understood as a consequence of insufficiently treatable pain. However, as core beliefs usually are formed early in life, probably before the start of migraine, the relationship between a core belief of helplessness and migraine is not clear. Either the negative core belief is transformed during life with disabling migraine to helplessness or patients with migraine and the core belief of helplessness develop a higher impact of their migraine pain. It could be speculated that imperatives around helplessness may reduce stress tolerance in relation to pain and thus promote the severity of the disease or the extent of the impairment by migraine. Further research on these topics would be highly interesting.

We see an unmet need for non-drug migraine preventive therapies. Neither do all migraineurs wish treatment with medication, nor do all qualify for or respond to the most advanced preventive treatments such as CGRP pathway antibody substances, which need to be applied regularly and are expensive. Once learned, a self-regulation method like Introvision can be applied individually, independent of time, place and situation. It is therefore also a cost-effective, sustainable, and supportive method with very limited side effects. Furthermore, stress reduction as the general aim of Introvision offers an additional benefit in daily life, especially as there is an epidemic of stress in our times.

## Conclusion

Although the primary endpoint was not reached, the results of the non-controlled part of the present study suggest that Introvision improves headache frequency and impact in migraineurs. In view of the paucity of data on non-medical interventions in migraine prevention, this is an important contribution to the field, which should prompt closer investigation of Introvision in a future randomized controlled study with improved design.

### Supplementary Information

**Additional file 1. **Introvision.

## Data Availability

The datasets used and analysed in the current study are available from the corresponding author on reasonable request.

## Abbreviations

SAP: Stating Attentive Perception
EG: Experimental Group
WL: Waiting List Group
HIT-6: Hedache-Impact-Test-6
FKMS: Fragebogen zum Kopfschmerzmanagment und zur Selbstwirksamkeit (german short form of the Headache Management Self-Efficacy Scale (HMSE))
MBSR: Mindfulness-based Stress Reduction
PMR: Progressive Muscle Relaxation
CGRP: Calcitonin-Gene Related Peptide
DMKG: Deutsche Migräne und Kopfschmerzgesellschaft, i.e. German Migraine and Headache Society
WPT: Wilcoxon-Paired Test

## References

[1] Goadsby PJ, Holland PR, Martins-Oliveira M, Hoffmann J, Schankin C, Akerman S (2017). Pathophysiology of Migraine: A Disorder of Sensory Processing. Physiol Rev.

[2] Demarquay G, Mauguière F (2016). Central Nervous System Underpinnings of Sensory Hypersensitivity in Migraine: Insights from Neuroimaging and Electrophysiological Studies. Headache.

[3] Meylakh N, Henderson LA (2022). Exploring alterations in sensory pathways in migraine. J Headache Pain.

[4] Van Casteren DS, Verhagen IE, Onderwater GL, Maassen VA, Terwindt G (2021). Sex differences in prevalence of migraine trigger factors: A cross-sectional study. Cephalalgia.

[5] Gossrau G, Zaranek L, Klimova A, Sabatowski R, Koch T, Richter M, Haehner A (2023) Olfactory training reduces pain sensitivity in children and adolescents with primary headaches. Front Pain Res 4:1091984 10.3389/fpain.2023.1091984 PMC9968932 36860330

[6] Fritsche G, Kröner-Herwig B, Kropp P, Niederberger U, Haag G (2013). Psychological therapy of migraine: systematic review. Schmerz.

[7] Buth B (2008). Die Anwendung von Introvision zur Verringerung der Belastung durch Tinnitus und Verbesserung der Hörfähigkeit. Gr Organ.

[8] Pereira Guedes N (2011) Dauerhafte Auflösung chronischer Nacken-/Muskelverspannungen durch Introvision: Eine empirische Untersuchung einer pädagogisch-psychologischen Intervention zur mentalen Selbstregulation. Thesis, University of Hamburg. https://ediss.sub.uni-hamburg.de/handle/ediss/3971

[9] Iwers-Stelljes TA, Plaum M, Oerding J (2012). Coaching in Introvision für weibliche Nachwuchs-führungskräfte: Erste Ergebnisse. Zeitschrift Organisationsberatung, Supervision, Coaching.

[10] Benthien O (2010) Stressreduktion im Leistungssport durch die pädagogisch-psychologische Methode der Introvision: eine theoretische und empirische Untersuchung am Beispiel des Segelsports. Thesis, University of Hamburg. https://ediss.sub.uni-hamburg.de/handle/ediss/4089. Accessed 22 Oct 2023

[11] Beck A, Rush T, John A (1987). Cognitive Therapy of Depression.

[12] Wagner AC (2011). Gelassenheit durch Auflösung innerer Konflikte. Mentale Selbstregulation und Introvision. 2nd Edition, Kohlhammer Verlag.

[13] Seminowicz DA, Burrowes SAB, Kearson A, Zhang J, Krimmel SR, Samawi L, Furman AJ, Keaser ML, Gould NF, Magyari T, White L, Goloubeva O, Goyal M, Peterlin BL, Haythornthwaite JA (2020). Enhanced mindfulness-based stress reduction in episodic migraine: a randomized clinical trial with magnetic resonance imaging outcomes. Pain.

[14] Herwig U, Kaffenberger T, Jäncke L, Brühl AB (2010). Self-related awareness and emotion regulation. Neuroimage.

[15] Olesen J (2018). Headache Classification Committee of the International Headache Society (IHS) the international classification of headache disorders. Cephalalgia.

[16] Kliem S, Mößle T, Zenger M, Brähler E (2014). Reliability and validity of the Beck depression Inventory-Fast Screen for medical patients in the general German population. J Affect Disord.

[17] Gandek B, Alacoque J, Uzun V, Andrew-Hobbs M, Davis K (2003). Translating the Short-Form Headache Impact Test (HIT-6) in 27 countries: methodological and conceptual issues. Qual Life Res.

[18] Graef JE, Rief W, French DJ, Nilges P, Nestoriuc Y (2015). German language adaptation of the Headache Management Self-Efficacy Scale (HMSE-G) and Development of a New Short Form (HMSE-G-SF). Headache.

[19] Wells RE, O'Connell N, Pierce CR, Estave P, Penzien DB, Loder E, Zeidan F, Houle TT (2021). Effectiveness of Mindfulness Meditation vs Headache Education for Adults With Migraine: A Randomized Clinical Trial. JAMA Intern Med.

[20] Grazzi L, D'Amico D, Guastafierro E, Demichelis G, Erbetta A, Fedeli D, Nigri A, Ciusani E, Barbara C, Raggi A (2023). Efficacy of mindfulness added to treatment as usual in patients with chronic migraine and medication overuse headache: a phase-III single-blind randomized-controlled trial (the MIND-CM study). J Headache Pain.

[21] Goadsby PJ, Reuter U, Lanteri-Minet M, da Silva P, Lima G, Hours-Zesiger P, Fernandes C, Wen S, Tenenbaum N, Kataria A, Ferrari MD, Klatt J (2021). Long-Term Efficacy and Safety of Erenumab: Results From 64 Weeks of the LIBERTY Study. Neurology.

[22] Meyer B, Keller A, Wöhlbier HG, Overath CH, Müller B, Kropp P (2016). Progressive muscle relaxation reduces migraine frequency and normalizes amplitudes of contingent negative variation (CNV). J Headache Pain.

[23] Kropp P, Meyer B, Dresler T, Fritsche G, Gaul C, Niederberger U, Förderreuther S, Malzacher V, Jürgens TP, Marziniak M, Straube A (2017). Relaxation techniques and behavioural therapy for the treatment of migraine : Guidelines from the German Migraine and Headache Society. Schmerz.

[24] Ruscheweyh R, Klonowski T, Goßrau G, Kraya T, Gaul C, Straube A, Jürgens TP, Scheidt J, Förderreuther S (2022). The headache registry of the German Migraine and Headache Society (DMKG): baseline data of the first 1,351 patients. J Headache Pain.

