# Supplementary material for: Effects of Introvision, a self-regulation method with a mindfulness-based perception technique in migraine prevention: a monocentric randomized waiting-list controlled study (IntroMig Study)
Source: J Headache Pain. 2023 Nov 3;24(1):146. doi: 10.1186/s10194-023-01684-0 (PMC10623798; doi:10.1186/s10194-023-01684-0)
Supplement: Supplementary file 1 — Additional file 1. Introvision. [file 10194_2023_1684_MOESM1_ESM.docx]

Supplementary Material

**Introvision**

Introvision is a mental and emotional self-regulation technique aiming to reduce stress and in-duce calm by resolving inner conflicts. It has been validated in over 40 years in a wide range of fields such as tinnitus^7^, chronic muscle tension^8^, mental blockages in female management trainees^9^, sleep quality, competitive sports^10^, and many more. According to the theory of Introvision, inner conflicts arise when individual core beliefs or inner demands, called „subjective imperatives“ collide with the perceived reality. Similar to trauma therapy, Introvision gradually enables to look calmly at the individual’s unpleasant feelings or anxieties.

According to Aaron Beck, negative core beliefs mainly circle around three conditions: feeling helpless, loveless, and/ or worthless^11^. If core beliefs such as „I must not be helpless“ are not met, the person may become stressed and agitated. The primary aim of Introvision is to detach this automatic link of negative emotion from the cognition.

In daily life, these individual core beliefs may be verbalized in rather banal self-instructions such as "I must always be on time" or "I must always look perfect". Accordingly, a person feels stressed or upset when the more superficial subjective imperative is not met by reality. In other words, a person with a core imperative manifesting as an everyday imperative "don't be late!" becomes stressed and tense realizing being late. The reason for being late is unsignificant, and also, it does not matter how hard the inner self tries to calm the mind with techniques such as rationalization ("everyone would be late if public transportation was out of service") or other individual calming techniques. These strategies may be useful in everyday life, but nevertheless are summarized in Introvision as „conflict avoidance strategies“: they are characterized by maintaining the immediate ability to act, but not solving the underlying problems. Sometimes this behavior is useful, but sometimes it becomes a habitualized, automated dysfunctional action strategy.

Introvision deals with the search for core beliefs, using the perception technique called "stating attentive perception" (SAP). SAP is a mindfulness-based, meditation-like method, aiming to reduce or eliminate perceptual filters. Applying SAP when internal conflicts arise, leads to a calming of agitation. Learning SAP begins with focusing visual, auditory, and sensory attention on a single object, sound, or body part respectively without being distracted by external or internal variables. Then the attention is expanded and focused without filters on the entire visual field, all audible sounds, or the entire body. In a next step, Introvision aims at directing attention simultaneously to the chosen focus and the entire range ("wide perception with constant focus") of a sensory modality. Finally, attention is directed to a mental cognition, and thoughts are stated in the focused and wide mode. After having learned to direct the attention intentionally in a wide and non-judging way to the described sense modalities, it is now directed to the mental level. First, attention is directed to the center of pleasantness and then to the center of unpleasantness. By mentally applying SAP in the wide-field mode, a person is enabled to look gradually and ever more calmly at his or her negative core beliefs. When the core belief is freed from agitation or the inner conflict is resolved, more serenity can be achieved in daily life. It becomes easier to see things as they are and thoughts of how things should be, diminish gradually^12^.

Previous studies have shown calming effects of mindfulness-based relaxation techniques and their effectiveness in migraine prevention ^6, 13^. Neurophysiologically it has been shown that perception techniques similar to Introvision quickly reduce the activity of the left amygdala, which explains the calming effect ^14^.
